# Assessing Workplace Bullying and Its Outcomes: The Paradoxical Role of Perceived Power Imbalance Between Target and Perpetrator

**DOI:** 10.3389/fpsyg.2022.907204

**Published:** 2022-06-14

**Authors:** Morten Birkeland Nielsen, Live Bakke Finne, Sana Parveen, Ståle Valvatne Einarsen

**Affiliations:** ^1^National Institute of Occupational Health, Oslo, Norway; ^2^Department of Psychosocial Science, University of Bergen, Bergen, Norway

**Keywords:** aggression, turnover, disempowerment, wellbeing, mistreatment

## Abstract

This study investigates the role of perceived power relation between target and perpetrator regarding victimization and turnover intent following exposure to bullying behavior at the workplace. We hypothesized that (1) targets of bullying behavior who self-label as victims experiences a larger power imbalance with the perpetrator compared to targets who do not self-label as victims, and (2) that the association between exposure to bullying behavior and intent to leave the job is stronger when there is power balance between target and perpetrator than when there is a power imbalance. The hypotheses were tested in a probability sample of employees working in the child welfare service in Oslo municipality, Norway, and that had been exposed to at least one instance of mistreatment from a colleague at their workplace (*N* = 374). Targets of bullying behavior whom self-labeled as victims reported a larger power imbalance with the perpetrator. Supporting the study hypothesis, and representing a reverse buffering effect, exposure to bullying behavior was most strongly associated with intent to leave among targets in power balance with the perpetrator. For targets in a perceived power imbalance, both low and high exposure to bullying behavior were associated with higher levels of intent to leave. These findings highlight the importance of implementing measures directed at preventing bullying and other forms of mistreatment, irrespective of the power relation between the two parties.

## Introduction

Workplace bullying refers to a situation where an employee is exposed to frequent harassing behavior from superiors or co-workers over a prolonged period of time and where the employee finds it difficult to defend him-/herself against these actions (Olweus, 1993; Einarsen and Skogstad, 1996). Following this description, workplace bullying represents a process with two distinct steps reflecting perceived “exposure” and “victimization.” In the “exposure” step, an employee experience systematic acts of aggression and mistreatment from others at the workplace over a prolonged period. These acts may range from relatively infrequent episodes of incivility to high-frequent exposure to harassment. In the “victimization” step, the exposed employee perceive him-/herself as unable to defend him-/herself against this exposure due to an experience of imbalance in power (Einarsen et al., 2011). As bullying, by definition, involves a victim-perpetrator relationship (Cuadrado-Gordillo, 2012), one may argue that this second step, the experience of disempowerment, is a prerequisite for describing a situation as bullying, and thereby differentiating bullying from “milder” forms of aggression such as incivility (Cowie et al., 2002; Saunders et al., 2007). However, in quantitative studies of those exposed, the aspect of power relation between the bullied and the bully often remains conceptual rather than empirical, as power relation is not explicitly measured when assessing bullying (Ciby and Raya, 2015; Nielsen et al., 2021). From a measurement methods perspective, this lack of overlap between the theoretical and operational definition of the bullying concept may contaminate the construct validity of research on bullying as it is likely that targets able to defend themselves against bullying should react differently compared to targets not able to defend themselves. In contrast, others have argued that measuring power relation explicitly is not necessary, as disempowerment is inherent in the assessment of bullying behaviors given the nature, prevalence, and duration of the ongoing mistreatment (Einarsen et al., 2009).

Due to these conflicting perspectives, there is a need for further empirical studies to determine the role of power relation in bullying (D'Cruz et al., 2018). To be able to make valid interpretations of the nature, frequency, antecedents, and outcomes of bullying, we need to be sure that our measurement instruments and assessment methods are sound and reliable. As the issue of power relation has been largely ignored in empirical studies on bullying to date, the aim of this study was to examine how power relation influences perceptions of workplace bullying and its outcomes. Specifically, by defining power relation as the degree of perceived psychological power imbalance between the bully and the bullied as seen from the perspective of the target, we will (1) examine whether power relation predicts victimization following exposure to bullying behavior and (2) investigate whether perceived power relation moderates the association between exposure to bullying behaviors and intent to leave. Previous research has established intent to leave as a main outcome of workplace bullying (Nielsen and Einarsen, 2012; Glambek et al., 2014; Spence Laschinger and Fida, 2014). Knowledge concerning moderators of how bullying influence intent to leave is therefore highly important regarding the development of interventions that can reduce the likelihood and costs of turnover in organizations.

### The Role of Power Relation in Workplace Bullying

A power imbalance between target and perpetrator may be present at the onset of the bullying behaviors or it might evolve and increase over a period (Ciby and Raya, 2015). In addition, the power imbalance could be either due to the formal power of organizational position or due to the informal power, such as, social support, knowledge and experience (Einarsen, 2000). Although a few qualitative studies have provided preliminary evidence for the importance of examining power relation (D'Cruz and Noronha, 2018; Patterson et al., 2018), there is a lack of quantitative research that can complement and extend the qualitative findings. To be able to understand why the aspect of power relation has received relatively little attention in quantitative investigations of workplace bullying, it is necessary to examine how bullying has been measured and assessed in existing research. To date, two main methods has been applied, each capturing different characteristics of the bullying phenomenon. The *behavioral experience method* assesses exposure to acts of harassment through an inventory that includes multiple types of unwanted and negative behavior typically experienced by victims of bullying, hence constituting exposure to severe bullying when occurring repeatedly over time. The respondents are then asked to report how frequently they have been exposed to the different behaviors listed in the inventory within a given time period (Einarsen et al., 2009; Nielsen et al., 2011). Accordingly, this method can be used to identify different forms of psychological harassment ranging from one-off incidences, such as incivility, to more severe and systematic harassment, such as exposure to on-going and severe workplace bullying and social exclusion. Hence, this method reflects the theoretical notion that bullying is a gradually escalating process that exist on a continuum rather than being an either-or phenomenon. While an important strength of the behavioral experience approach is that the method allows for assessing the nature, frequency, and duration of the unwanted behaviors that characterize bullying, power relation between target and perpetrator is not explicitly measured (Nielsen et al., 2011, 2017). Consequently, the method does not permit inferences about experienced disempowerment.

The second approach used to assess workplace bullying is the *self-labeling method* (Nielsen et al., 2011). Here, respondents are given a single-item question, often accompanied by a theoretical definition of bullying, asking about whether they have perceived themselves as bullied within a specific time-period (e.g., Einarsen and Skogstad, 1996; O'Moore et al., 2003). This method has high face validity, is efficient in terms of applying only one question to measure the phenomenon and is easy to administer, yet also treating exposure to bullying more as an end state where one is either a victim of bullying or not. However, from a psychometric point of view, using single-item measures is often discouraged as such measures allegedly suffer from reliability issues (Gardner and Cummings, 1998). Furthermore, the method does not offer nuanced insights in the actual behaviors involved in the bullying, nor does it provide any information regarding the severity or persistency of these behaviors. Finally, although the self-labeling method allows for determining whether the respondent feels victimized by bullying, it does not provide any explicit information about the perceived power relation with the perpetrator, apart from it often being a part of the definition that accompanies the question about bullying. Hence, findings based on this method are restricted to whether the respondents perceive themselves as *victims* of bullying, and is a highly subjective approach likely to be biased by a range of factors such as personality, emotional states, cognitions, and misperceptions (Nielsen et al., 2011). Compared to the behavioral experience approach, prevalence rates tend to be lower in studies employing this assessment method (Ilies et al., 2003; Nielsen et al., 2010), thus indicating that the threshold for labeling oneself as a victim of bullying is high.

Taken together, a limitation of existing assessment methods is that they do not provide direct and explicit information regarding the power relation between target and perpetrator (Ciby and Raya, 2015). However, knowledge about power relation is necessary to understand the nature, causes, and outcomes of bullying. According to Salin (2003), bullying is a specific type of workplace aggression as the target is placed in a helpless and defenseless position. An imbalance in power on behalf of the target regarding the perpetrator is therefore considered as a main aspect of the definition of workplace bullying (Cowie et al., 2002; Einarsen et al., 2011). This victim-perpetrator structure indicates that a perceived power imbalance, be it formal or informal, is a prerequisite for bullying to occur, and victimization following exposure to bullying behavior should therefore only take place if the target is in power imbalance with the perpetrator. Without having information about the power relation when assessing bullying, we do not know if the target would be able to withstand the negative acts and even retaliate, thus preventing these negative acts to escalate into bullying. An implication is that the target should perceive him-/herself as disempowered in order to self-label as a victim. To test this assumption, we propose the following hypothesis.

*H1: Targets of bullying behavior that self-label as victims experiences a larger power imbalance with the perpetrator compared to targets who do not self-label as victims*.

Since power imbalance is such a central aspect of definitions of workplace bullying, it seems reasonable to expect that any health and wellbeing outcome of being exposed to bullying behaviors are determined by the power relation between the bully and the bullied. However, different theoretical perspective provides different explanations for how power relation influences the outcomes of bullying and, depending on the theoretical perspective, it can be argued that being in power balance with the perpetrator can both *lessen* and *amplify* the negative effects of being exposed to bullying (Nielsen et al., 2017). Consequently, exactly how power relations influence the outcomes of bullying is still not clear. In the following section the two contrasting perspectives are described.

The first perspective is centered around classical work stress models, with the well-established *Transactional model of stress and coping* (Lazarus and Folkman, 1984) as the main basis, and suggests that being in power balance with the perpetrator should attenuate the negative effects of bullying. According to the Transactional model, the nature and severity of reactions following exposure to a given stressor are functions of a dynamic interplay between event characteristics and individual appraisal and coping processes. When a person is faced with a stressor, the person evaluates the potential threat (primary appraisal) and a judgment is made as to whether the event is positive or negative (Lazarus, 1993). As a secondary appraisal, the person evaluates how controllable the stressor is and determines whether ones available coping resources are adequate for handling and mastering the situation (Lazarus and Folkman, 1984). Therefore, if the target perceives him-/herself as being in power balance with the perpetrator, the target will be more likely to handle the situation and the perceived bullying should have less impact. On the other hand, if the target is in power imbalance with the perpetrator, he/she should be unable to handle and control the exposure, which could lead to a state of fatalism and resignation. Being exposed to repeated and enduring painful or otherwise aversive stimuli that the targeted person is unable to escape or avoid has been shown to be related to health impairment (Maier and Seligman, 2016). Hence, according to this first perspective, targets of bullying who are in power imbalance with the perpetrator should be more likely to experience detrimental consequences, including health problems and reduced wellbeing, than should targets who are in a balanced power relation with the perpetrator.

Contrasting this view, the potential amplifying effects of being in power balance with the perpetrator is derived from the *Behavioral incongruence hypothesis*. The Behavioral incongruence hypothesis suggest that individuals experience negative affect when they engage in behaviors that are incompatible with their personality preferences or self-concepts (Diener et al., 1984; Ilies et al., 2011). That is, it is assumed that a person will experience more positive and less negative affect when there is congruence between a given situation and their preferences (Pervin, 1993). In contrast, individuals will experience heightened negative affect in situations that are incompatible with their personality preferences (Diener et al., 1984; Ilies et al., 2011). Unemployment may serve as an illustration for this process. Most people want to work, and unemployed people are thereby being caught in a life situation that they do not want to be in. This state of incongruence will lead to negative affect and is therefore possibly a cause of higher levels of mental health problems found among unemployed (Paul and Moser, 2006). This line of reasoning can be extended to workplace bullying. For a target of bullying in a balanced power relation with the perpetrator, relatively infrequent and short term spells of mistreatment, such as incivility (Cortina et al., 2001), should be harmless since the perceived power balance secures an overall pervasive and enduring feeling of confidence with regard to managing the situation (Demsky, 2019). On the other hand, long-lasting and systematic exposure to severe harassment will be especially detrimental for targets perceiving themselves to be in power balance with the perpetrator since such treatment is unanticipated and creates a pervasive feeling of dissonance in the target. That is, for targets who perceive themselves as being in a balanced power relation, being repeatedly exposed to harassment over a long period of time is likely to lead to an incongruity between their self-perceptions of being able to withstand bullying, and how they actually are treated by the bullies.

Feelings of violation or incongruence have been proposed to be the byproduct of a sensemaking process where the individual attempts to derive meaning from a perceived breach of obligation (Morrison and Robinson, 1997), in this case on the part of a colleague at the workplace. The discrepancy between expected and actual events stimulates information seeking, explanation, and interpretation and people are particularly likely to seek sensemaking information when events are unexpected and negative and when they appear in relational exchange relationships (Morrison and Robinson, 1997; Sears and Humiston, 2015). As we need consistency in our conceptual system, unsuccessful sensemaking, i.e., unresolved incongruence, may be experienced as deeply shattering and may subsequently result in psychological distress, reduced wellbeing, and thereby an increased motivation for leaving one's job (Janoff-Bulman, 1992; Mikkelsen and Einarsen, 2002). Based on this perspective, it is therefore likely that perceiving oneself to be in power balance with the perpetrator, while not being able to withstand and stop the unwanted mistreatment, leads to a state of incongruence that will amplify the effects following exposure to harassment. In contrast, for targets who perceive themselves to be in imbalance with the perpetrator, exposure to bullying behavior will serve as a confirmation of the unbalanced power relation and thereby lead to a feeling of congruence between expectations and actual experience.

To our knowledge, only one quantitative study to date to have examined the impact of power relation on the outcomes following exposure to bullying behavior (Nielsen et al., 2017). In support of the behavioral incongruence hypothesis, the findings showed that power balance (measured as “ability to defend” with a single item question) only had a protective effect on the relationship between exposure to bullying behaviors and target anxiety level in cases of very low exposure. In cases of high exposure, there was a stronger increase in levels of anxiety among employees reporting being able to defend themselves than among those who generally felt unable to defend themselves. To extend the findings by Nielsen et al. (2017), the current study will examine the interactive effects of power relation regarding associations between exposure to bullying behaviors and a job-related outcome in the form of intent to leave. Based on the conclusions from the abovementioned study, in conjunction with the behavioral incongruence hypothesis, we find it most plausible to expect the relationship between bullying and intent to leave to be most detrimental among targets that perceive themselves to be in power balance with the perpetrator, whereas those who are in power imbalance already want to leave their job and thereby do not report any changes turnover intent in cases of high frequent exposure to bullying behavior. Thus, the following hypothesis will be tested.

*H2: There is a stronger (positive) association between exposure to bullying behavior and intent to leave the job when there is a power balance between target and perpetrator than when there is a power imbalance*.

## Methods

### Design and Sample

The data were collected as part of the “Oslo Workplace Aggression Survey” (OWAS), a collaborative project between the Norwegian National Institute of Occupational Health (STAMI) and the vice mayor of education and child services in Oslo municipality. The survey was conducted electronically in March 2020. All employees (*N* = 1,264) working full or part time in the child welfare service in Oslo municipality received an email with an invitation to participate in a survey in which the employees were asked to fill in an anonymous self-reporting questionnaire assessing exposure to threats and violence, workplace bullying and conflicts, different aspects of the psychosocial working environment, work stress, and health and wellbeing. The data collection division of the Norwegian National Institute of Occupational Health was responsible for the sampling procedures, implementation, and quality assurance. As all employees in the organization were invited to participate, the sampling approach can be described as a probability procedure (Ilies et al., 2003). To ensure anonymity in the data collection, the researchers, were not informed about names, addresses, or other identifying information. A further description of the project and its background is provided in a separate project protocol (Nielsen et al., 2020a).

A total of 678 questionnaires were returned, yielding a response rate of 53.6%. The sample consisted of 74.4% women and 25.6% men. The mean age was 39 years (SD = 10.91). A total of 82.4% worked in a full-time position, 10.4% in a part-time position, while 6.6% were on-call staff. 0.6% were on temporary leave. Altogether 16.6% of the respondents had some sort of formal leadership responsibility.

As the overarching aim of this study was to examine the role of power relation *when exposed to harassing behavior* at the workplace, the study sample was limited to respondents who reported exposure to at least one bullying behavior in the employed Negative Acts Questionnaire Revised and the questions about power relation (*N* = 374). Exposure to at least one behavior was chosen as inclusion criterion since there is no universally agreed upon cut-off criterion for when an exposure constitutes bullying. The subsample did not differ from the overall sample regarding demographic characteristics.

### Ethical Approval and Consent to Participate

The project was conducted in accordance with the World Medical Association Declaration of Helsinki. The Regional Committees for Medical and Health Research Ethics in Norway (REC South East) have approved the project (project number 28496). In line with the General Data Protection Regulation (GDPR), the National Institute of Occupational Health acquired permission from the Norwegian Center for Research Data (NSD; approval: 226309) to process the personal data in this project for research purposes. When accessing the web-based questionnaire by a personal login code, the respondents had to confirm their informed consent before responding to the questionnaire. This procedure for securing informed consent was approved by the ethics committee and NSD. No personally identifiable information about respondents were available to the researchers, as data were de-identified prior to analyses.

### Instruments

All questionnaire items used in this study are included in **Appendix 1.**

The nine-items *Short Negative Acts Questionnaire* (S-NAQ) was used to measure perceived exposure to specific *bullying behaviors at the workplace* (Notelaers et al., 2018). Among others, the S-NAQ covers behavior such as being withheld information, being excluded or humiliated and being given unmanageable workloads The S-NAQ has previously been validated against other bullying measures, as well as measures of health, sickness absence, work performance and turnover intention (Einarsen et al., 2009). The respondents were asked how often they had been exposed to the behavior during the last 6 months, with response categories on a 5-point frequency scale ranging from 1 = “never,” 2 = “occasionally,” 3 = “monthly,” 4 = “weekly,” to 5 = “daily” (e.g., “If you look back over the past 6 months how often did it happen that people insulted you?”). The S-NAQ had a Cronbach's alpha value of 0.85 in the present study. By measuring harassment using the S-NAQ, where the response alternatives range from occasional and monthly incidences to weekly and daily events, we were able to examine the full spectrum of harassing behavior, going from incivility to bullying. That is, although the distinction between incivility and bullying behavior may be blurry (Hershcovis, 2011), thus making it difficult to determine when incivility develops into bullying behavior, we can be sure that the response categories of the S-NAQ capture both low-frequent and high frequent episodes of harassment.

*Power relation* between target and perpetrator as seen from the target's perspective was assessed with a three-item scale developed for this study as an add-on to the S-NAQ. Directly following the S-NAQ, the respondents were asked: “If you have been exposed to one or more of the behaviors in the list above, did you…” (1)”…experience it as difficult to defend yourself against this treatment?,” (2) “…experience a feeling of hopelessness and resignation in relation to what you have been exposed to,” and (3) “…feel inferior and powerless in relation to the person or persons who performed the actions.” Response alternatives were “never,” “sometimes,” “once in a while,” “often,” and “every time.” Higher scores indicate that the target is in power imbalance with the perpetrator. Cronbach's alpha for the power relation scale was 0.91.

*Victimization from workplace bullying* was measured with the well-established self-labeling method (Olweus, 1991; Einarsen and Skogstad, 1996; Solberg and Olweus, 2003; Nielsen et al., 2011). After being presented with the following definition: “Bullying (harassment, badgering, niggling, freezing out, offending someone) is a problem in some workplaces and for some workers. To label something bullying it must occur repeatedly over a period of time, and the person confronted has to have difficulties defending himself/herself. It is not bullying if two parties of approximately equal “strength” are in conflict or the incident is an isolated event,” respondents were asked “*Have you been subjected to bullying at the workplace during the last 6 months?*” The response categories were “no,” “rarely,” “now and then,” “once a week,” and “several times a week.” In this study, positive responses, i.e., “rarely” to “several times a week” were recoded into a single “self-labeling” category.

*Turnover intentions* were measured with a three-item questionnaire (Sjoberg and Sverke, 2000), each item being assessed by the respondents on a five-point Likert scale ranging from “fully disagree” to “fully agree.” The scale measures searching for new jobs (e.g., “I am actively searching for a new job”) as well as willingness to quit given an adequate alternative (e.g., “If I had a free choice, I would quit this job”). Cronbach's alpha for the scale was 0.90.

### Control Variables

Age, gender, and leadership responsibility were included as control variables in all multivariate analyses. Although existing evidence is inconclusive, studies have established age differences (De Cuyper et al., 2009) and gender differences (Glambek et al., 2018) with regard to outcomes of workplace bullying. Having a leadership position is associated with formal power in an organization and is therefore likely to influence the power relation in cases of bullying.

### Data Analyses Plan

Statistical analyses were conducted with IBM SPSS 27.0 and MPLUS 8.4. The level of significance was set to *p* < 0.05. For all measurement inventories, summary scales were calculated based on a mean-score of their respective items. Independent sample *t*-tests and logistic regression analysis was used to examine group difference in power relation regarding self-labeled victimization. Cohen's *d* and Hedges' *g* were calculated to determine the magnitude of the group differences (effect size). Cohen (1988) suggested using the following rule of thumb for interpreting results: Small effect; 0.2, medium effect: 0.5, large effect: 0.8. Hedges' *g* is interpreted using the same thresholds. To explore main and moderating effects, we conducted a hierarchical regression analysis with the PROCESS 4.0 script in SPSS (Hayes, 2012) to test for linear associations between exposure to bullying behaviors and intent to leave, as well as the interactive effects of exposure to bullying and power relation, with regard to intent to leave. The guidelines by Baron and Kenny (1986) were followed, and, in line with Aiken and West (1991), the continuous predictor variables were centered prior to the two-way interaction analysis.

## Results

Table 1 presents the descriptive statistics of the study variables and their zero-order correlations. All correlations were in the expected directions. As the constructs are theoretically assumed to represent second order constructs of the bullying phenomenon, it was not surprising that the correlation between exposure to bullying behavior and power imbalance were high (*r* = 0.63; *p* < 0.001). To determine whether measurement instruments were empirically different, we followed a confirmatory approach with MPLUS that compared a one-factor measurement model (i.e., all items loading on a “bullying” factor) with a two-factor solution (i.e., items loading separately on “exposure to bullying behavior” and “power relation”) using the study sample of 374 respondents. The analyses showed that the two-factor solution (X2 = 153.868; df = 53; CFI = 0.99; TLI = 0.98; RMSEA = 0.07; 95% CI RMSEA = 0.06 – 0.08) had significantly better fit to the data (ΔX2 = 26.589; df = 1; *p* < 001) when compared to the one-factor model (X2 = 180.457; df = 54; CFI = 0.98; TLI = 0.98; RMSEA = 0.08; 95% CI RMSEA = 0.07 – 0.09). All factor loadings exceeded 0.40 with no cross-loadings or error correlations. The findings confirm that exposure to bullying behavior and power relation represent separate and unique aspects of the bullying constructs.

**Table 1:** Descriptive statistics and intercorrelations for all study variables (*N* = 374).

	**Variable**	**M**	**SD**	**1**	**2**	**3**	**4**	**5**	**6**	**7**
1.	Gender	0.74	0.44	–						
2.	Age	38.19	11.03	−0.03	–					
3.	Leadership position	0.15	0.36	0.08	0.36***	–				
4.	Bullying behavior	1.21	0.31	0.07	0.01	0.04	–			
5.	Power relation	1.56	0.86	0.16***	−0.01	−0.01	0.63***	–		
6.	Intent to leave	2.42	1.23	0.05	−0.11*	−0.06	0.28***	0.27**	–	
7.	Self-labeled bullying	1.06	0.25	0.06	−0.02	0.02	0.41***	0.30**	0.06	–

*p < 0.05; **p < 0.01; ***p < 0.001.

*Coding for gender: Males: 0, Females: 1. Coding for leadership position: Non-leaders: 0, Leaders: 1*.

In total, 6.4% of those exposed to bullying behavior self-labeled as victims of bullying. An independent sample *t*-test was conducted to test the hypothesis that targets who self-labels as victims of bullying experiences a larger power imbalance with the perpetrator than to targets who do not self-label as victims. The findings supported the hypothesis. Those who self-labeled as victims (M = 2.54; SD = 1.17) reported significantly (*t* = −6.06; df = 372; *p* < 0.001) larger power imbalance with the perpetrator compared to targets who did not self-label (M = 1.49; SD = 0.79). Estimates of effect size (Cohen's *d* = 1.05; Hedges' *g* = 1.28) showed that the group difference was large and substantive. A logistic regression analysis was conducted to determine the mutual impact of exposure to negative acts and power relation on self-labeled victimization. Exposure to negative acts was significantly exposed to increased risk of self-labeling as a victim (OR = 12.88; 95% CI = 3.70 – 44.86). Although at the borderline to significance, power relation was not significantly associated with self-labeling in this analysis (OR = 1.56; 95% CI = 0.99 – 2.47). There was no evidence for an interactive effect of exposure to negative acts and power relation regarding self-labeling as a victim (OR = 1.67; 95% CI = 0.53 – 5.87). These findings show that power relation does not contribute to the variance over and above exposure to bullying behavior, thus indicating that the aspect of power imbalance is implicitly embedded in the exposure.

**Figure 1:**
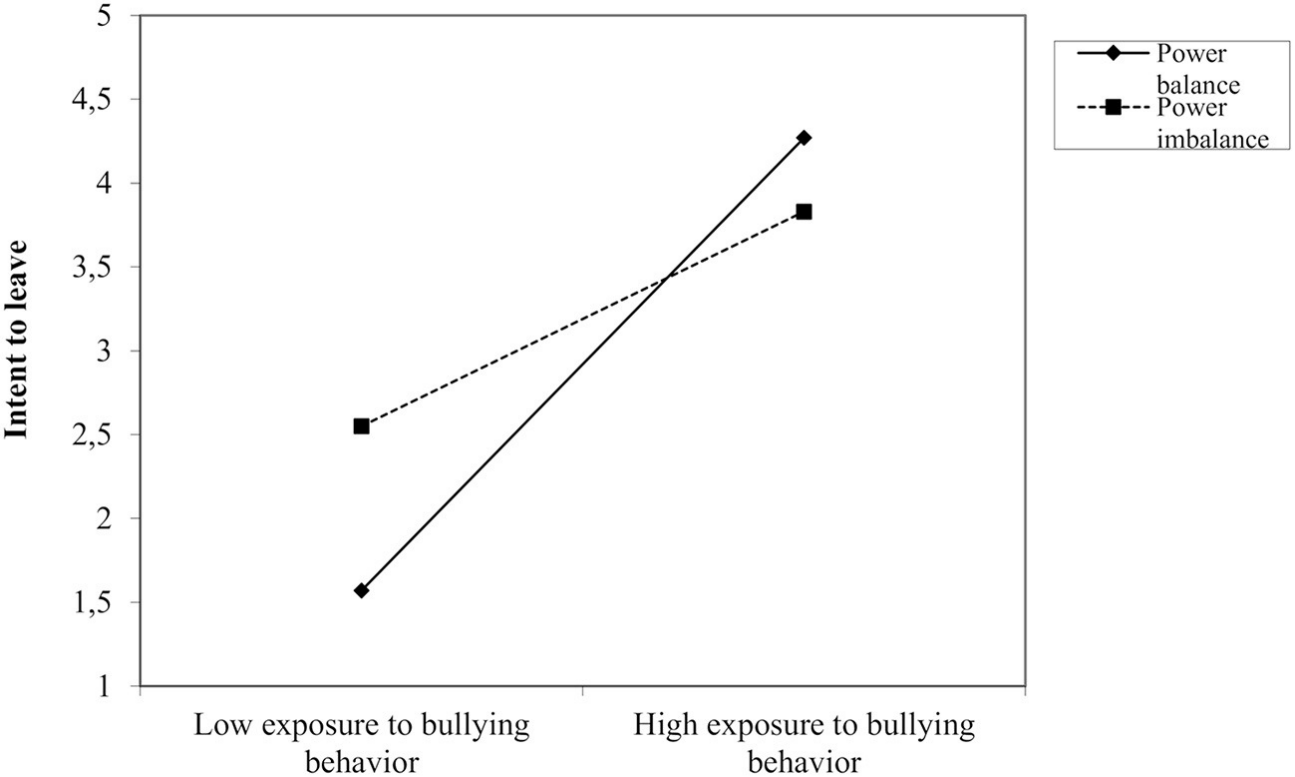
Interaction between exposure to bullying behaviors and power relation regarding intentions to leave.

A hierarchical regression analysis was conducted to test the main and moderating effects of exposure to bullying behavior and power relation regarding intent to leave (Table 2). Adjusting for gender, age, and leadership position, we found that both bullying behaviors (β = 0.33; *p* < 0.001) and power relation (β = 0.19; *p* < 0.01) had significant positive main effects on intent to leave. The interaction term between bullying behavior and power relation was also significant (β = −0.15; *p* < 0.001). To examine the nature of this interaction, scores were plotted at the mean, low (1 SD below the mean) and high (1 SD above the mean) values on the indicators of bullying behavior and power relation. In support of the second hypothesis, the findings showed a stronger association between bullying behavior and intent to leave among respondents who perceived themselves to be in power balance with the perpetrator (β = 0.44; *p* < 0.001), than among respondents in power imbalance (β = 0.20; *p* < 0.01). That is, while respondents in power imbalance with the perpetrator report high levels of intent to leave irrespective of the frequency of the bullying behavior, respondent in power balance report an increase in intent to leave as the frequency of bullying behavior increases (see Figure 1).

**Table 2:** Exposure to bullying behavior and power relation as predictors of intent to leave (main and interactive effects).

	**B**	**SE B**	**95 CI**	**β**
Gender	0.02	0.16	−0.29 – 0.01	0.02
Age	−0.01	0.01	−0.02 – 0.01	−0.01
Leadership position	−0.16	0.21	−0.57 – 0.26	−0.12
Bullying behavior	1.35	0.34	0.67 – 2.02	0.33***
Power relation	0.27	0.10	0.07 – 0.47	0.19**
Bullying behavior × power relation	−0.71	0.20	−1.10 – −0.33	−0.15***
Constant	2.92	0.38	2.17 – 3.67	0.40**

*R2 = 0.14; F = 7.83 df = 6/292; p <0.001. R2 change = 0.04; p < 0.001*.

*p < 0.05; **p < 0.01; ***p < 0.001.

The above analyses were replicated after removing the control variables age, gender, and leadership position. The findings from these supplementary analyses were consistent with the main analyses. This suggests that the controls appear to have no effect on the inferences.

## Discussion

We found power relation with the perpetrator to be a determinant of both self-labeled victimization from bullying and intentions to leave when exposed to bullying behaviors while at work. In support of our first hypothesis, targets of bullying behavior that self-labeled as victims experienced more power imbalance with the perpetrator when compared to targets who did not perceive themselves as victims. Supporting our second hypothesis, we found a stronger association between exposure to bullying behavior and turnover intent among respondents who perceived themselves to be in power balance with the perpetrator compared to those who reported to be in power imbalance. Specifically, the findings pointed to a reverse buffering effect where power balance with the perpetrator only had a protective effect on intent to leave in cases of low exposure to bullying behavior. In cases of high exposure, targets in power balance reported equal levels of intent to leave as targets in power imbalance. All levels of exposure to bullying were associated with high intent to leave among respondents who perceived themselves to be in power imbalance with the perpetrator. As we discuss below, our theory and findings offer insights on how individuals may respond to workplace bullying differently depending on their power relation with the perpetrator. We also discuss the implications of our findings for future research and practices.

### Implications for Theory and Future Research

Although most theoretical definitions of workplace bullying highlight the role of power imbalance between target and perpetrator (Einarsen et al., 2011), the issue of power relation has been largely ignored in the measurement of the bullying construct (Nielsen et al., 2011; Ciby and Raya, 2015). The findings of this study challenge the current tradition of not including power relation in assessment of bullying. First, self-labeled victims of bullying report larger power imbalance with the perpetrator than those that do not self-label. Second, as shown by our factor analysis, exposure to bullying behavior and power relation were empirically distinct constructs and should therefore be measured separately. Yet, their intercorrelations were rather high, indicating that higher exposure to bullying behavior is associated with increased power imbalance. This finding can be interpreted in two ways. One possible interpretation is that those in power imbalance is likely to be easier targets and therefore also report more exposure. This is in line with the notion of “predatory bullying” which refers to cases where the target has done nothing provocative that may reasonably justify the behavior of the bully, but where the perpetrator either is demonstrating power or in other ways is trying to exploit an accidental victim into compliance (Einarsen et al., 2017). Alternatively, high exposure may alter the experience of power (im-)balance over time. In such cases, power imbalance may be a result of the process development of bullying which starts with exposure and reaches its final stage when the target self-labels as a victim (Einarsen, 2000).

We also found that power relation did not contribute to the variance in self-labeling as a victim over and above exposure to bullying behavior. This could denote that the aspect of power imbalance is embedded in the mistreatment in cases of high frequency exposure to bullying behavior. Especially considering that it is unlikely for a target to be exposed to systematic aggression at a weekly or daily basis without being in an actual power imbalance with the perpetrator. An implication of this finding is that it may not be necessary to add an explicit measure of power relation when assessing the severe episodes of bullying. However, as shown by our findings on the outcomes of exposure to bullying behaviors, power relation plays a crucial role in cases of low intensity exposure. Specifically, although power relation may have less impact regarding the outcomes following high intensity exposure to bullying behavior, assessing power relation when examining more low-intensity forms of mistreatment, such as workplace incivility, is highly important. If the aspect of power relation is excluded from assessment of outcomes following such low-intensity forms of workplace mistreatment, one risks losing important nuanced information concerning how mistreatment takes place and affects those exposed. A noteworthy paradox is that the role of power relation has received little attention in research on workplace incivility to this date (Demsky, 2019).

The somewhat counterintuitive finding that exposure to bullying behaviors had a stronger association with intent to leave among targets who perceive themselves to be in power balance with the perpetrator supports the Behavioral incongruence hypothesis (Diener et al., 1984; Ilies et al., 2011) and is in line with a previous study showing that bullying behavior was most strongly associated with anxiety among targets that perceived themselves to be able to defend themselves against the perpetrator (Nielsen et al., 2017). The finding also corresponds with a series of studies showing that personal characteristics such as sense of coherence (Nielsen et al., 2008), agreeableness (Ilies et al., 2011), and coping styles (Reknes et al., 2016) only have a protective effect with regard to outcomes in cases of no or only low exposure to bullying behaviors at the workplace. In cases of high exposure, bullying behaviors seem to be detrimental for all. Interestingly, this finding goes against well-established theoretical models on stress, such as the Transactional model of stress and coping (Lazarus and Folkman, 1984) and the Cognitive activation theory of stress and coping (Ursin and Eriksen, 2004), which both suggest that individual capacities will act as protective resources with regard to stressor-strain relationships. This may indicate that workplace bullying represents an especially detrimental stressor that exceeds other psychosocial hazards at the workplace. However, as this study was limited to intent to leave as an outcome, future research on workplace bullying should examine the role of power relation regarding more health-related outcomes such as anxiety, depression, and somatic complaints. As discussed in the introduction, understanding the role of the sensemaking process among targets of bullying may be especially important.

### Prevention and Policy Implications

Across assessment methods, it has been estimated that about 15% of employees are subjected to workplace bullying at any time (Nielsen et al., 2010). Systematic reviews and meta-analyses of cross-sectional and longitudinal evidence show that bullying is associated with increased mental health problems (Nielsen and Einarsen, 2012; Verkuil et al., 2015), physical complaints (Nielsen et al., 2014), suicidal ideation (Leach et al., 2017), sleep problems (Nielsen et al., 2020b), and reduced work ability (Nielsen et al., 2016). The findings from this study complement this line of evidence by showing that even low intensity exposure to bullying behaviors at the workplace can be detrimental for those exposed, at least in cases where the targets perceive themselves to be in power imbalance with the perpetrator. In cases of escalated exposure, bullying is detrimental for all.

As bullying represent both a prevalent and harmful psychosocial hazard at the workplace, an up-front implication concerns the importance of developing effective human resource strategies to prevent and handle bullying in organizations. Moreover, it is essential that these new strategies also apply to, and take into consideration, how one should deal with even less intense cases of bullying. As being exposed to systematic bullying behaviors are experienced as problematic even for employees in power balance with the perpetrator, organizations and employers must actively intervene in the early stages of the bullying process rather than believing that the targeted worker is robust enough to be able to deal with the exposure him-/herself (Nielsen et al., 2017). Organizational efforts focusing on primary interventions, such as building a strong psychosocial safety climate (Bond et al., 2010; Law et al., 2011) or a strong climate for constructive conflict management (Einarsen et al., 2017, 2018) may be the most effective way to prevent workplace bullying from occurring and harming employees and the organizations. For those investigating cases of bullying, the nature of the power relation between the involved parties should be considered even in cases of less systematic exposure. The inventory presented in the current study may be one way of assessing power relations.

### Methodological Strengths and Limitations

Important strengths of the current study are applying a probability sample and the relatively high response rate. The indicators of bullying and intent to leave were valid and well-established instruments. In addition, the current study also provides evidence for the validity of the tool developed to measure power relation. We note two main limitations of this research. First, the study is based on cross-sectional data, a design that does not allow for causal inferences. Although we have based the study on the theoretical assumption that bullying is a precursor to turnover intent with power relation as a moderator, other associations are also possible. For instance, it may be that employees who expresses turnover intentions are more likely to be bullied because they are perceived as disloyal by their colleagues and that this leads to an experience of power imbalance. The study variables should therefore be further examined using longitudinal data. Nonetheless, the knowledge that many pairs of variables are associated, even without knowing their causal connections, is extremely valuable as a basis for theory and the target of intervention (Spector, 2019).

Second, all data were collected using self-report questionnaires, which could hamper the internal validity of the findings. For instance, there is the possibility of subjective interpretations, common method variance and response set tendencies (Spector, 2006). However, as both bullying and intent to leave have subjective components and are influenced by perceptions, assessing these phenomena by using objective methods is difficult. Several steps were taken to reduce problems associated with common-method variance, including varying response anchors for different subscales, ensuring that the independent variables were presented in different sections of the survey from the dependent variable, and emphasizing to participants that their responses would be anonymous (Podsakoff et al., 2003).

It should be mentioned that the prevalence of self-labeled victimization was rather low in the current study (6%). Also, the levels of exposure to bullying behaviors were rather low. While these rates are in line with previous findings on prevalence in Norway (Nielsen et al., 2009), it is well-established that the prevalence rates of bullying in Norway are rather low compared to other countries (Van de Vliert et al., 2013). To validate the findings, this study should therefore be replicated in a country or culture with higher prevalence rates or employees may be more accepting of power inequalities and abusive supervision.

## Conclusion

Theoretically, a real or perceived power imbalance between target and perpetrator is a necessary condition for labeling mistreatment at the workplace as bullying. However, our research shows that power relation has a counterintuitive role in cases of bullying behavior at the workplace. While power imbalance does seem to be a determinant of victimization following exposure to bullying behaviors, its impact on the outcomes following bullying is somewhat more paradoxical, at least when seen from the perspective of classical stress theories. That is, exposure to high-frequent mistreatment at the workplace appears to be detrimental for all, irrespective of whether the target perceive him-/herself as being in a balanced power relation with the perpetrator. Experiencing a balanced power relation with the perpetrator seem to only be beneficial in cases of low-intensity mistreatment. This indicates that workplace bullying represents a highly demanding and detrimental workplace stressor. A consequence is that organizational leaders should be concerned with implementing measures directed at preventing bullying or any systematic mistreatment for all employees, not only where there is an imbalance in their power relation.

## Data Availability Statement

The raw data supporting the conclusions of this article will be made available by the authors, without undue reservation.

## Ethics Statement

The studies involving human participants were reviewed and approved by the Regional Committees for Medical and Health Research Ethics in Norway (REC South East). The patients/participants provided their written informed consent to participate in this study.

## Author Contributions

MN in initiated the study, conducted analyses, and was responsible for writing the manuscript. SE and LF participated in the idea development, contributed to the structure and content, and read all versions of the manuscript. SP was responsible for the data collection, participated in the idea development, contributed to the structure and content, and read all versions of the manuscript. All authors contributed to the article and approved the submitted version.

## Conflict of Interest

The authors declare that the research was conducted in the absence of any commercial or financial relationships that could be construed as a potential conflict of interest.

## Publisher's Note

All claims expressed in this article are solely those of the authors and do not necessarily represent those of their affiliated organizations, or those of the publisher, the editors and the reviewers. Any product that may be evaluated in this article, or claim that may be made by its manufacturer, is not guaranteed or endorsed by the publisher.

## Appendix 1. Questionnaire Items in This Study

### Short Negative Acts Questionnaire

Response categories: “Never,” “Now again,” “Monthly,” “Weekly,” “Daily.”

1 Someone withholding information which affects your performance.

2 Spreading gossip and rumors about you.

3 Being ignored or excluded.

4 Having insulting or offensive remarks made about your person, attitudes or your private live.

5 Being shouted at or being a target of spontaneous rage.

6 Repeated reminders of your errors or mistakes.

7 Being ignored or facing a hostile reaction when you approach.

8 Persistent criticism of your work and effort.

9 Practical jokes carried out by people you do not get along with.

### Power Relations in Bullying Inventory

Instructions: This inventory should follow directly after the items in the Negative Acts Questionnaire or another behavioral experience inventory assessing workplace bullying.

Response categories: “Never,” “Sometimes,” “Once in a while,” “Often,” “Every time.”

“If you have been exposed to one or more of the behaviors in the list above, did you…”

1. … experience it as difficult to defend yourself against this treatment?

2. … experience a feeling of hopelessness and resignation in relation to what you have been exposed to?

3. …feel inferior and powerless in relation to the person or persons who performed the actions?

### Intent to Leave

Response categories: “Totally disagree,” “Disagree,” “Neither disagree nor agree,” “Agree,” “Totally agree.”

1. I am actively looking for other jobs.

2. I feel that I could leave this job.

3. If I was completely free to choose, I would leave this job.

## References

[B1] Aiken L. S. West S. G. (1991). Multiple Regression: Testing and Interpreting Interactions. Newbury Park, CA: Sage.

[B2] Baron R. M. Kenny D. A. (1986). The moderator-mediator variable distinction in social psychological research: conceptual, strategic, and statistical considerations. J. Personal. Soc. Psychol. 51, 1173–1182. 10.1037/0022-3514.51.6.1173 3806354

[B3] Bond S. A. Tuckey M. R. Dollard M. (2010). Psychosocial safety climate, workplace bullying, and symptoms of posttraumatic stress. Org. Dev. J. 28, 28–37.

[B4] Ciby M. Raya R. P. (2015). Workplace bullying: a review of the defining features, measurement methods and prevalence across continents. IIM Kozhikode Soc. Manag. Rev. 4, 38–47. 10.1177/2277975215587814

[B5] Cohen J. (1988). Statistical Power Analysis for the Behavioral Sciences. Hillsdale, NJ: Lawrence Earlbaum Associates.

[B6] Cortina L. M. Magley V. J. Williams J. H. Langhout R. D. (2001). Incivility in the workplace: incidence and impact. J. Occup. Health Psychol. 6, 64–80. 10.1037/1076-8998.6.1.64 11199258

[B7] Cowie H. Naylor P. Rivers I. Smith P. K. Pereira B. (2002). Measuring workplace bullying. Aggr. Violent Behav. 7, 33–51. 10.1016/S1359-1789(00)00034-3

[B8] Cuadrado-Gordillo I. (2012). Repetition, power imbalance, and intentionality: do these criteria conform to teenagers' perception of bullying? A role-based analysis. J. Interperson. Violence 27, 1889–1910. 10.1177/0886260511431436 22203634

[B9] D'Cruz P. Noronha E. (2018). Abuse on online labour markets: targets' coping, power and control. Qualit. Res. Org. Manag. 13, 53–78. 10.1108/QROM-10-2016-1426

[B10] D'Cruz P. Noronha E. Lutgen-Sandvik P. (2018). Power, subjectivity and context in workplace bullying, emotional abuse and harassment: insights from postpositivism. Qualit. Res. Org. Manag. 13, 2–9. 10.1108/QROM-12-2017-1587

[B11] De Cuyper N. Baillien E. De Witte H. (2009). Job insecurity, perceived employability and targets' and perpetrators' experiences of workplace bullying. Work Stress 23, 206–224. 10.1080/02678370903257578

[B12] Demsky C. A. (2019). Unpacking the role of power in incivility. Indus. Org. Psychol. 12:81. 10.1017/iop.2019.81

[B13] Diener E. Larsen R. J. Emmons R. A. (1984). Person x Situation interactions: choice of situations and congruence response models. J. Personal. Soc. Psychol.47:6491870. 10.1037/0022-3514.47.3.580 6491870

[B14] Einarsen K. Mykletun R. J. Einarsen S. V. Skogstad A. Salin D. (2017). Ethical infrastructure and successful handling of workplace bullying. Nordic J. Working Life Stud. 7, 37–53. 10.18291/njwls.v7i1.81398

[B15] Einarsen S. (2000). Harassment and bullying at work: a review of the Scandinavian approach. Aggr. Violent Behav. 5, 379–401. 10.1016/S1359-1789(98)00043-3

[B16] Einarsen S. Hoel H. Notelaers G. (2009). Measuring exposure to bullying and harassment at work: validity, factor structure and psychometric properties of the Negative Acts Questionnaire-Revised. Work Stress 23, 24–44. 10.1080/0267837090281567332286111

[B17] Einarsen S. Hoel H. Zapf D. Cooper C. L. (2011). “The concept of bullying and harassment at work: the European tradition,” in Bullying and Harassment in the Workplace. Developments in Theory, Research, and Practice, 2nd Edn, eds, S. Einarsen, H. Hoel, D. Zapf and C. L. Cooper (Boca Raton, FL: CRC Press), 3–40. 10.1201/EBK1439804896-3

[B18] Einarsen S. Skogstad A. (1996). Bullying at work: epidemiological findings in public and private organizations. Eur. J. Work Org. Psychol. 5, 185–201. 10.1080/13594329608414854

[B19] Einarsen S. Skogstad A. Rørvik E. Lande Å. B. Nielsen M. B. (2018). Climate for conflict management, exposure to workplace bullying and work engagement: a moderated mediation analysis. Int. J. Hum. Resour. Manag. 29, 549–570. 10.1080/09585192.2016.1164216

[B20] Gardner D. G. Cummings L. L. (1998). Single-item versus multiple-item measurement scales: an empirical comparison. Educ. Psychol. Measur. 58, 898–915. 10.1177/0013164498058006003

[B21] Glambek M. Matthiesen S. B. Hetland J. Einarsen S. (2014). Workplace bullying as an antecedent to job insecurity and intention to leave: a 6-month prospective study. Hum. Resour. Manag. J. 24, 255–268. 10.1111/1748-8583.12035

[B22] Glambek M. Nielsen M. B. Gjerstad J. Einarsen S. (2018). Gender differences in the relationship between workplace bullying and subjective back and neck pain: a two-wave study in a Norwegian probability sample. J. Psychosomat. Res. 106, 73–75. 10.1016/j.jpsychores.2018.01.010 29455903

[B23] Hayes A. F. (2012). PROCESS: A Versatile Computational Tool for Observed Variable Mediation, Moderation, and Conditional Process Modeling. Available online at: http://www.afhayes.com/public/process2012.pdf (accessed May 2, 2022).

[B24] Hershcovis M. S. (2011). “Incivility, social undermining, bullying… oh my!:” a call to reconcile constructs within workplace aggression research. J. Org. Behav. 32, 499–519. 10.1002/job.689

[B25] Ilies R. Hauserman N. Schwochau S. Stibal J. (2003). Reported incidence rates of work-related sexual harassment in the United States: using meta-analysis to explain reported rate disparities. Person. Psychol. 56, 607–631. 10.1111/j.1744-6570.2003.tb00752.x

[B26] Ilies R. Johnson M. D. Judge T. A. Keeney J. Johnson M. D. O. (2011). A within-individual study of interpersonal conflict as a work stressor: dispositional and situational moderators. J. Org. Behav. 32:677. 10.1002/job.677

[B27] Janoff-Bulman R. (1992). Shattered Assumptions. Towards a New Psychology of Trauma. New York, NY: The Free Press.

[B28] Law R. Dollard M. F. Tuckey M. R. Dormann C. (2011). Psychosocial safety climate as a lead indicator of workplace bullying and harassment, job resources, psychological health and employee engagement. Accid. Anal. Prev. 43, 1782–1793. 10.1016/j.aap.2011.04.010 21658506

[B29] Lazarus R. S. (1993). Coping theory and research - past, present, and future. Psychosom. Med. 55, 234–247. 10.1097/00006842-199305000-00002 8346332

[B30] Lazarus R. S. Folkman S. (1984). Stress, Appraisal and Coping. New York, NY: Springer.

[B31] Leach L. S. Poyser C. Butterworth P. (2017). Workplace bullying and the association with suicidal ideation/thoughts and behaviour: a systematic review. Occup. Environ. Med. 74, 72–79. 10.1136/oemed-2016-103726 27663985

[B32] Maier S. F. Seligman M. E. P. (2016). Learned helplessness at fifty: insights from neuroscience. Psychol. Rev. 123:rev0000033. 10.1037/rev0000033 27337390 PMC4920136

[B33] Mikkelsen E. G. Einarsen S. (2002). Basic assumptions and symptoms of post-traumatic stress among victims of bullying at work. Eur. J. Work Org. Psychol. 11, 87–11. 10.1080/13594320143000861

[B34] Morrison E. W. Robinson S. L. (1997). When employees feel betrayed: A model of how psychological contract violation develops. Acad. Manag. Rev. 22:259230. 10.2307/259230

[B35] Nielsen M. B. Christensen J. O. Hetland J. Finne L. B. (2020a). Organizational prevention and management strategies for workplace aggression among child protection workers: a project protocol for the Oslo Workplace Aggression Survey (OWAS). Front. Psychol. 11:1401. 10.3389/fpsyg.2020.01401 32695050 PMC7339981

[B36] Nielsen M. B. Einarsen S. (2012). Outcomes of workplace bullying: a meta-analytic review. Work Stress 26, 309–332. 10.1080/02678373.2012.734709

[B37] Nielsen M. B. Gjerstad J. Jacobsen D. P. Einarsen S. V. (2017). Does ability to defend moderate the association between exposure to bullying and symptoms of anxiety? Front. Psychol. 2017:1953. 10.3389/fpsyg.2017.01953 29163321 PMC5682040

[B38] Nielsen M. B. Harris A. Pallesen S. Einarsen S. V. (2020b). Workplace bullying and sleep - a systematic review and meta-analysis of the research literature. Sleep Med. Rev. 51:101289. 10.1016/j.smrv.2020.101289 32179375

[B39] Nielsen M. B. Indregard A. M. Øverland S. (2016). Workplace bullying and sickness absence – a systematic review and meta-analysis of the research literature Scand. J. Work Environ. Health 42, 359–370. 10.5271/sjweh.3579 27310716

[B40] Nielsen M. B. Magerøy N. Gjerstad J. Einarsen S. (2014). Workplace bullying and subsequent health problems. Tidsskrift for den Norske legeforening 134, 1233–1238. 10.4045/tidsskr.13.0880 24989201

[B41] Nielsen M. B. Matthiesen S. B. Einarsen S. (2008). Sense of coherence as a protective mechanism among targets of workplace bullying. J. Occup. Health Psychol. 13, 128–136. 10.1037/1076-8998.13.2.128 18393582

[B42] Nielsen M. B. Matthiesen S. B. Einarsen S. (2010). The impact of methodological moderators on prevalence rates of workplace bullying. A meta-analysis. J. Occup. Org. Psychol. 83, 955–979. 10.1348/096317909X481256

[B43] Nielsen M. B. Notelaers G. Einarsen S. (2011). “Measuring exposure to workplace bullying,” in Bullying and Emotional Abuse in the Workplace. Developments in Theory, Research and Practice, eds S. Einarsen, H. Hoel, D. Zapf and C. L. Cooper (Boca Raton, FL: CRC Press), 9. 10.1201/EBK1439804896-9

[B44] Nielsen M. B. Notelaers G. Einarsen S. V. (2021). “Methodological issues in the measurement of workplace bullying,” in Bullying and Harassment in the Workplace. Theory, Research and Practice, 3rd Edn, eds. S.V. Einarsen, H. Hoel, D. Zapf and C. L. Cooper (Boca Raton, FL: CRC Press), 235–268. 10.1201/9780429462528-8

[B45] Nielsen M. B. Skogstad A. Matthiesen S. B. Glaso L. Aasland M. S. Notelaers G.. (2009). Prevalence of workplace bullying in Norway: comparisons across time and estimation methods. Eur. J. Work Org. Psychol. 18, 81–101. 10.1080/13594320801969707

[B46] Notelaers G. Van der Heijden B. Hoel H. Einarsen S. (2018). Measuring bullying at work with the short-negative acts questionnaire: identification of targets and criterion validity. Work Stress 33, 58–75. 10.1080/02678373.2018.1457736

[B47] Olweus D. (1991). “Bullying/victim problem among school children,” in The Development and Treatment of Childhood Aggression, eds I. Rubin and D. Pepler. (Hillsdale, NJ: Erlbaum), 1.

[B48] Olweus D. (1993). Bullying at Schools: What We Know and What We Can Do. Oxford: Blackwell.

[B49] O'Moore M. Lynch J. Niamh n.D. (2003). The rates and relative risks of workplace bullying in Ireland, a country of high economic growth. Int. J. Manag. Decision Mak. 4, 82–95. 10.1504/IJMDM.2003.002490

[B50] Patterson E. Branch S. Barker M. Ramsay S. (2018). Playing with power: examinations of types of power used by staff members in workplace bullying - a qualitative interview study. Qualit. Res. Org. Manag. 13, 32–52. 10.1108/QROM-10-2016-1441

[B51] Paul K. I. Moser K. (2006). Incongruence as an explanation for the negative mental health effects of unemployment: meta-analytic evidence. J. Occup. Org. Psychol. 79, 595–621. 10.1348/096317905X70823

[B52] Pervin L. A. (1993). Personality. Theory and Research. New York, NY: John Wiley & Sons, Inc.

[B53] Podsakoff P. M. MacKenzie S. B. Lee J. Y. Podsakoff N. P. (2003). Common method biases in behavioral research: a critical review of the literature and recommended remedies. J. Appl. Psychol. 88, 879–903. 10.1037/0021-9010.88.5.879 14516251

[B54] Reknes I. Einarsen S. Pallesen S. Bjorvatn B. Moen B. E. Magerøy N. (2016). Exposure to bullying behaviors at work and subsequent symptoms of anxiety: the moderating role of individual coping style. Indus. Health Adv. 54, 421–432. 10.2486/indhealth.2015-0196 27151548 PMC5054283

[B55] Salin D. (2003). Ways of explaining workplace bullying: a review of enabling, motivation and precipitating structures and processes in the work environment. Hum. Relat. 56, 1213–1232. 10.1177/00187267035610003

[B56] Saunders P. Huynh A. Goodman-Delahunty J. (2007). Defining workplace bullying behaviour professional lay definitions of workplace bullying. Int. J. Law Psychiatr. 30, 340–354. 10.1016/j.ijlp.2007.06.007 17692375

[B57] Sears K. Humiston G. S. (2015). The role of emotion in workplace incivility. J. Manag. Psychol. 30:373. 10.1108/JMP-11-2012-0373

[B58] Sjoberg A. Sverke M. (2000). The interactive effect of job involvement and organizational commitment on job turnover revisited: a note on the mediating role of turnover intention. Scand. J. Psychol. 41, 247–252. 10.1111/1467-9450.00194 11041307

[B59] Solberg M. E. Olweus D. (2003). Prevalence estimation of school bullying with the Olweus Bully/Victim Questionnaire. Aggr. Behav. 29, 239–268. 10.1002/ab.10047

[B60] Spector P. E. (2006). Method variance in organizational research - truth or urban legend? Org. Res. Methods 9, 221–232. 10.1177/1094428105284955

[B61] Spector P. E. (2019). Do not cross me: optimizing the use of cross-sectional designs. J. Bus. Psychol. 34, 125–137. 10.1007/s10869-018-09613-8

[B62] Spence Laschinger H. K. Fida R. (2014). A time-lagged analysis of the effect of authentic leadership on workplace bullying, burnout, and occupational turnover intentions. Eur. J. Work Org. Psychol. 23, 739–753. 10.1080/1359432X.2013.804646

[B63] Ursin H. Eriksen H. R. (2004). The cognitive activation theory of stress. Psychoneuroendocrinology 29, 567–592. 10.1016/S0306-4530(03)00091-X 15041082

[B64] Van de Vliert E. Einarsen S. Nielsen M. B. (2013). Are national levels of employee harassment cultural covariations of climato-economic conditions? Work Stress 27, 106–122. 10.1080/02678373.2013.760901

[B65] Verkuil B. Atasayi S. Molendijk M. L. (2015). Workplace bullying and mental health: a meta-analysis on cross-sectional and longitudinal data. PLoS ONE. 10:e135225. 10.1371/journal.pone.0135225 26305785 PMC4549296

